# Prediction of novel drug indications using network driven biological data prioritization and integration

**DOI:** 10.1186/1758-2946-6-1

**Published:** 2014-01-07

**Authors:** Ala Qabaja, Mohammed Alshalalfa, Eisa Alanazi, Reda Alhajj

**Affiliations:** 1 Department of Computer Science, University of Calgary, Calgary, Alberta, Canada; 2 Department of Computer Science, University of Regina, Regina, Canada; 3 Biotechnology Research Center, Palestine Polytechnic University, Hebron, Palestine; 4 Biotechnology Department, An-Najah University, Nablus, Palestine; 5 Computer Science Department, Global University, Beirut, Lebanon; 6 Department of Computer Science, Umm Al-Qura University, Makkah, Saudi Arabia

**Keywords:** Disease, Drug, Gene, Protein networks

## Abstract

**Background:**

With the rapid development of high-throughput genomic technologies and the accumulation of genome-wide datasets for gene expression profiling and biological networks, the impact of diseases and drugs on gene expression can be comprehensively characterized. Drug repositioning offers the possibility of reduced risks in the drug discovery process, thus it is an essential step in drug development.

**Results:**

Computational prediction of drug-disease interactions using gene expression profiling datasets and biological networks is a new direction in drug repositioning that has gained increasing interest. We developed a computational framework to build disease-drug networks using drug- and disease-specific subnetworks. The framework incorporates protein networks to refine drug and disease associated genes and prioritize genes in disease and drug specific networks. For each drug and disease we built multiple networks using gene expression profiling and text mining. Finally a logistic regression model was used to build functional associations between drugs and diseases.

**Conclusions:**

We found that representing drugs and diseases by genes with high centrality degree in gene networks is the most promising representation of drug or disease subnetworks.

## Background

The development of many methods that enable the isolation and study of individual cells and molecules has revolutionized the process of drug discovery from being at the physiological level to more the accurate molecular level. This revolution was all due to the genome sequencing project that provides a complete list of genes and gene products and enables the simultaneous monitoring of the expression of the whole genome. Consequently, this technology has shed light on possible computational techniques for investigating new therapeutic applications for already approved drugs or other safe drug candidates in what is called drug repositioning. By definition, drug repositioning techniques ignore the first testing phases, that might take a decade and cost more than 1$ billion, and progresses directly to drug applications [1]. This strategy certainly has the potential of being the most efficient technique for drug discovery since it provides reduced development costs and shorter paths to approval [2].

Computational prediction of drug-disease associations has become one of the leading approaches to drug-disease treatment investigation. Network and systems biology enable a better understanding for drug discovery by considering a global physiological environment of protein targets. Thus network biology has played a central role in developing efficacious therapies that alter entire pathways rather than single proteins, resulting in the potential for fighting complex multifactorial diseases [3]. This finding confirms that medicine is no exception to the mathematical system theory that states the scale and complexity of the solution should match the scale and complexity of the problem. It seems clear that therapies modulating a single target yield nothing but minor alteration of a diseases complex machinery. Therefore for the past few years the focus to fight complex diseases has been on network centric but not gene centric [4] modules. Out of the different approaches and data sources that have been used for drug repositioning, microarrays and text mining have been the most prevalent. Gene expression microarrays have been broadly and successfully used to study the molecular pathophysiology of diseases [5-8] and drug mode of action [9-12]. Noteworthy that most of these approaches were based on gene set enrichment (GSEA) statistical techniques [13]. For instance, Lamb et al. [10] studied hundreds of molecules over different cell lines, drug doses and exposure time slots. This approach has enabled Lamb and colleagues to create ranked lists of genes for each sample and finally to use GSEA to build associations from different molecules. Similarly Iorio et al. [12] used a merging procedure to merge all the ranked lists related to a particular drug into one representative ranked list of genes for the drug. Finally they applied GSEA to build a drug-drug network based on the same concept. On the other hand, there were many attempts to prioritize disease-associated genes by integrating microarray expression profiles and network data [14-17]. As described by Wu et al. [17] these techniques can be sorted into three major categories. The first uses microarray data and t-tests to find possible differentially expressed genes (DEG). Later on it uses a gene network in order to prioritize genes that are surrounded by DEGs [14]. The second technique considers the dynamic changes in interactions of the candidate gene with other genes in the compared samples (normal and disease samples), which has been done by defining hubs from a protein-protein interaction network (PPIN) and checking if the hub and its neighbors are co-expressed together in different tissues [15]. The third technique considers variations of gene interactions between compared samples and their effects on gene expression to prioritize disease-associated genes [16]. More specifically, it defines the set of DEG together with a manually curated set of transcription regulators (TRs). Later, the difference in coexpression between DEGs and TRs is computed in the compared conditions. This difference is used for computation of differential wiring that is going to be used for prioritization purposes.

**Figure 1:**
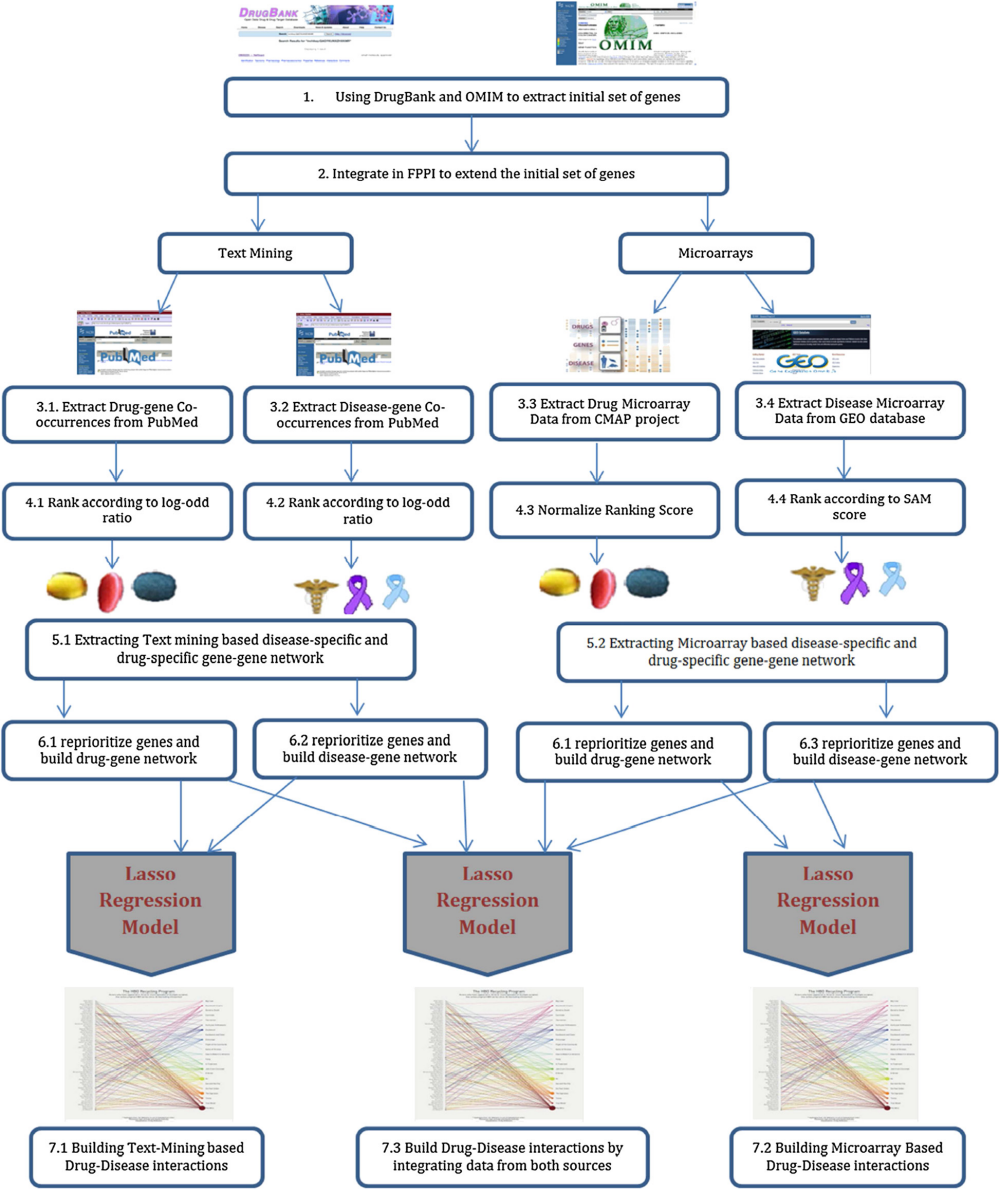
**General framework for building drug-disease associations.** This figure shows the general framework for our proposed paradigm. Steps 1 and 2 were used to extract the initial set of genes. Steps 3.1 and 3.2 extracted drug-gene and disease-gene co-occurrences, respectively. Steps 3.3 and 3.4 extracted drug microarray and disease microarray data respectively. In step 4 we found the ranks of genes related to a specific drug or disease. In step 5 we built the drug-specific and disease-specific gene-gene network. In step 6 we ran prioritization procedures as to launch drug-gene and disease-gene interaction networks into a lasso regression. In step 7 we used lasso regression model to build drug-disease associations.

In addition to microarray expression profiles, many text mining based tools and biological systems have been successfully developed to connect and prioritize genes, diseases and drugs. Some of these approaches use pattern-based recognition techniques [18] and others integrate protein-protein networks for prioritization purposes [19-21]. For instance, Cheng et al. [18] have developed a web-based text mining system called PolySearch for extracting relationships between human diseases, genes, mutations, drugs and metabolites. PolySearch employs a text ranking scheme to score the most relevant sentences and abstracts that associate both the query and match terms with each other. Li et al. [19] proposed a paradigm that integrates molecular interaction network mining and text mining techniques. The proposed paradigm starts by incorporating disease-specific seed genes/proteins derived from prior knowledge. This seed of genes is improved by expanding and re-ranking them in the functional context by reprioritizing them in disease-related molecular interaction networks. To avoid the problem of being biased towards the initial set of genes, OzgÃijr et al. [21] developed a framework that integrates a text-mining curated protein-protein network that is related to a particular disease with social network analysis centrality measures to predict unknown disease-gene associations. The authors used sentence parsing in order to build a syntactic parse tree representing the syntactic constituent structure of a sentence and to build a protein-protein network from this tree. After building the disease specific protein-protein network, the authors considered all the seed genes in addition to their neighbors for further analysis. Finally to prioritize genes related to a particular disease, they used degree, eigenvector, betweenness and closeness network centrality metrics. It is noteworthy that some chemical structure similarity approaches were used in drug repositioning in addition to the text-mining and microarray based approaches. An outstanding paper in this field was the one by Gottlieb et al. [22]. Their approach was designed to directly predict drug-disease associations including both FDA approved drugs and other molecules in the experimental phase. Their algorithm works in three phases: (i) building five drug-drug similarity measures and two disease-disease similarity measures; (ii) building classification features and subsequent learning classification rule that can distinguish between true and false drug-disease associations by using these similarity measures; and (iii) applying a logistic regression classifier to predict any new possible drug-disease associations. Thus for a given drug-disease association from the gold standard (experimentally curated list of drug-disease interactions), the authors computed an association score by considering all the other known drug-disease association. Even though this technique attained high sensitivity and specificity in cross-validation experiments, it is not without limitations. Firstly, the proposed method used 5 different drug similarity measures and 3 different disease similarity measures. This makes it biased to include a drug without having its chemical structure, side effects, target sequence, target PPIN and its target gene ontology. The same thing is applicable to diseases. Furthermore this method does not consider the similarity between drugs molecular actions. It only considers the known targets for drugs to define similarities. Sometimes there might be hidden or unknown drug targets that are not considered in this study resulting in a bias since drugs trigger their action on target genes and have a consequent effect on other off-target genes. From the approaches described above, one can conclude that microarray expression profiles mining, text mining and biological network analysis are very robust techniques when it comes to connecting biological entities (drugs, diseases and genes). In this work, we will study, analyze and try to predict drug-disease associations in a more contextualized view that is provided by network biology. This strategy will propel their association from the classical empiricism to a pathway-based rational design of global therapies. The main goal of this work is to identify a set of genes that are prioritized according to their relevancy to a particular disease or drug and then use these associations to build a drug-disease association network. Thus instead of identifying disease related or drug related genes from an expert-curated source, we will be utilizing text mining and microarray data as genes associated with a complex disease pathway are not all identified. Moreover, many of these genes and proteins are still under investigation for potential values as disease biomarkers. The initial set of genes extracted from each source will be further extended from a source specific network, once by utilizing a microarray based network and the other by utilizing a text-mining based network, by including their direct neighbors for further analysis. By doing this network-based approach, each drug or disease will be represented by a subnetwork where edges represent an interaction and nodes represent the set of seed genes for that particular drug/disease and their direct neighbors. Later on, genes from each subnetwork will be reprioritized according to their centrality measures in that subnetwork. Finally a lasso regression model is used to predict drug-disease associations by using drug-gene and disease-gene interaction networks using three different sources: microarray data, text-mining data and finally an integrative source that combines information from these two sources. A general description for the proposed method is described in Figure 1.

## Methods

### Defining the initial set of genes

In an experiment to quantitatively assess the druggable potential of the human genome, conceivable results indicated that only 10% of the genes in human genome are considered drug targets, 10% are involved in disease pathophysiology and only 5% are both druggable and relevant to disease [23]. We assumed that including only genes that are related to a drugs mode of action or a diseases pathophysiology can save processing time and memory by excluding irrelevant genes from further analysis. Particularly, we used DrugBank database [24] to include all targets of our drug set and OMIM database to include genes that are involved in disease pathophysiology.

### Refining gene lists using protein networks

To guarantee that we had selected a robust function set of genes for drugs and diseases, we included other functionally related genes, thereby extending our understanding for drug mode of action or disease pathophysiology. For this purpose we used functional protein interactions from Reactome database [25] in order to extract all the other genes that are functionally related (direct neighbors) to our seed list of genes. In the context of this work, we will refer to these lists as DiseaseExt and DrugExt for the extended lists of diseases and drugs, respectively.

### Prioritizing genes using microarray and text mining data

In this section we describe two directions we followed to prioritize DiseaseExt and DrugExt genes. In the first approach, we used microarray expression data of cells treated with drugs and diseases to rank genes based on their differential expression capability. In the second approach we used text mining techniques to rank genes based on the frequency of their co-occurrence with diseases or drugs.

#### Prioritizing gene lists based on microarray gene expression

Two different databases were used to generate microarray based drug-gene and disease-gene interactions. For drugs, we used the Connectivity Map website [26] that contains 6100 ranked lists of genes for 1300 chemical substances. Note that ranking scores for genes are based on their differential expression between untreated and drug treated samples. So for a set of n genes the most positively expressed gene was given a rank of 1 and the most negatively expressed gene was given a rank of n. We extracted these ranked lists and merged repeated samples for a particular drug as has been described by Iorio [12]. Thus we ended up having a representative list of each of the remaining 406 drugs after excluding chemical substances that are not recognized in the DrugBank database [24]. We extracted the rank values for DrugExt genes and normalized rank scores for each gene relevant to a particular drug according to this list. Finally the 25 lowest and the 25 highest ranked genes for each drug were selected to represent the initial set of genes to a build drug-specific gene network. We will refer to these sets as Mir-DrugExt. For diseases, we used the Gene Expression Omnibus (GEO) repository to generate microarray data for disease samples and control samples. To select datasets, it was essential in this experiment to select disease expression profiles that were generated using Human Affymetrix platform to make it consistent with the experiments generated for drugs and avoid any possible platform-specific bias. Also it was essential to include a set of diseases with.CLE raw files uploaded since we planned to normalize experiments with the same normalization algorithm. This set of diseases was selected by manually browsing GEO for disease experiments that satisfy the mentioned criteria. This browsing process was done by two bioinformaticians and lasted for two weeks, resulting in a set of 24 diseases. CLE files for the 24 diseases were collected independently and RMA normalization algorithm [27] was used in order to normalize data. We extracted the gene expression profiles for the DiseaseExt gene set by finding a corresponding probe-set in microarray expression profiles. Note that, the average expression profiles for all probe-sets have been taken for genes that are represented by more than one corresponding probe-set. Later on, we used significant analysis of microarray or SAM technique [28] in order to identify a differential expression score for every single gene, and the genes were ranked according to their scores from 1 to n. SAM assigns a score based on changes related to standard deviations of some randomly generated measurements of a particular gene. This score ranges between a high positive indicating that the gene has been up-regulated upon comparison between healthy and diseased samples, and a high negative score indicating that the gene has been down-regulated upon comparison between healthy and diseased samples. Finally the 25 lowest and 25 highest ranked genes for each disease were selected to represent the initial set of genes to build a disease-specific gene network. We will refer to these sets as Mir-DiseaseExt.

#### Prioritizing gene lists based on PubMed abstracts

To prioritize genes for each drug and disease, we found co-occurrences between these biological entities using PubMed abstracts. More specifically, we queried PubMed database to check the co-occurrences of every single disease/drug with every single gene in DiseaseExt and DrugExt. It is noteworthy that we considered all possible annotations or MeSH terms for specific disease, drug or gene. Since co-occurrences can be vulnerable to false positives, we set to zero any drug-gene or disease-gene co-occurrence that was less than 5. After defining these co-occurrences we used regularized a log odd ratio connectivity measure to reflect the strength of the ties in our drug-gene and disease-gene co-occurrence matrices. The resulting score yielded a positive value for enriched drug-gene or drug-gene pairs and a negative value for underrepresented pairs. As described previously [19], the connectivity between a particular drug or disease D and a gene G or *Connect*_*DG*_ can be computed according to the following formula:

(1) $${\text{Connect}}_{\text{DG}}=\text{ln}({\text{ABS}}_{\text{DG}}*N+\lambda )-\text{ln}({\text{ABS}}_{G}*{\text{ABS}}_{D}+\lambda )$$

Where *ABS*_*DG*_ is the total number of abstracts in which drug or disease D and gene G were co-mentioned together. *ABS*_*G*_ and *ABS*_*D*_ is the number of abstracts in which gene G and drug or disease D was mentioned, respectively. N is the size of all tested abstracts. *λ* is a small constant that has been added to avoid out of bound errors in case any of *ABS*_*DG*_, *ABS*_*G*_, or *ABS*_*D*_ values were zero. The only concern with using this formula is that the N term in our case is very big (all abstracts in PubMed), thus making score biased toward the left hand side of the formula. On the other hand, using any small reasonable value to replace N would make the score biased toward the right hand side of the formula. Therefore we sought to modify both sides to fit our analysis according to the following formula:

(2) $$\begin{array}{ll} \;{\text{Connect}}_{\text{DG}}= & \text{ln}({\text{ABS}}_{\text{DG}}*\text{max}({\text{ABS}}_{D},{\text{ABS}}_{G})+\lambda )\\ & -\text{ln}({\text{ABS}}_{D}+{\text{ABS}}_{G}+\lambda ) \end{array}$$

And we set *λ* to 1 in all cases. Finally we included all genes with a positive *Connect*_*DG*_ score relevant to a particular disease or drug. We will refer to these gene sets as Txt-DiseaseExt and Txt-DrugExt for diseases and drugs respectively.

### Generating disease-specific and drug-specific gene module signatures

**Figure 2:**
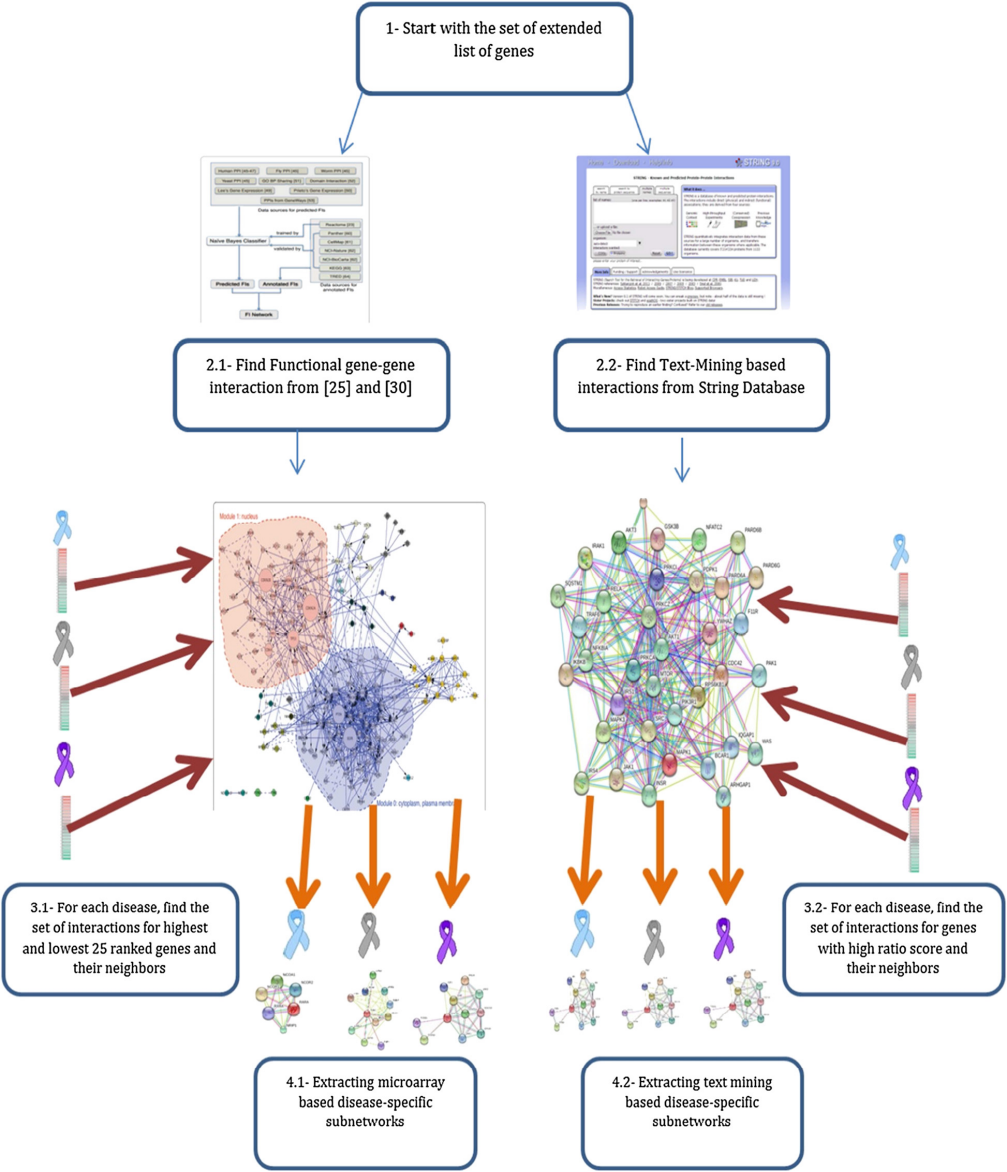
**Generating a disease-specific and drug-specific gene-gene network.** This figure shows the process of generating the drug-specific and disease-specific gene-gene network. The process starts by finding all possible interactions between the initial set of genes in steps 2.1 and 2.2 both from a text-mining source and a functional PPI source, respectively. The initial list of genes for each disease and each drug are then used to query the extracted network in a data source specific manner. Finally the interactions between these genes and their direct neighbors would be considered as a disease-specific or drug-specific gene-gene network.

After we refined the DiseaseExt and DrugExt gene sets using microarray and text mining techniques, we sought to find gene subnetworks (gene modules) to represent each drug and disease. As described above, our major goal was to utilize the information that is stored in biological networks and thus focus our attention on network topological features to predict drug indications. We intended to generate two subnetworks for every single drug or disease using two different sources of information: microarray expression profiles (Mir-DiseaseExt and Mir-DrugExt) and text mining data (Txt-DiseaseExt and Txt-DrugExt). To generate the text-mining based subnetworks we first extracted a comprehensive network that represents gene-gene interactions from text papers. More specifically we used the whole set of genes we were working on to query STRING web server [29]. STRING server stores a huge gene-gene network derived from four different sources: genomic context, high throughput technology, co-expression and text mining. We extracted text mining based interactions between Txt-DiseaseExt and Txt-DrugExt genes for each disease and each drug respectively. We will use the terms TxtNet-DiseaseExt and TxtNet-DrugExt to refer to these interactions. We used a similar methodology in order to generate the microarray based subnetworks. The only difference was with generating the comprehensive network that represents the interactions between all genes. Since microarrays measure the level of expression between genes and can be utilized to understand functional relationship between genes, we sought to use a functional gene-gene network to generate microarray based network. For this purpose, we extracted the interactions between our set of genes (Mir-DiseaseExt and Mir-DrugExt) both from a functional protein-protein network [25] and a signaling network [30]. We will use MirNet-DiseaseExt and MirNet-DrugExt to refer to functional protein interactions specific to each disease and drug respectively. The whole process of generating disease-specific subnetworks is described in Figure 2. Note that everything in Figure 2 applies to finding drug-specific subnetworks.

### Using logistic regression to build drug-disease associations

Logistic regression measures the relationship between a binary response variable (Disease-gene network) and one or more predictor variables (drug-gene networks). We used logistic regression modeling in this work as the response variables, which represent association between diseases and genes, are binary. To model this problem as a regression model, we write the disease gene network as a linear combination of the drug-gene subnetworks. In other words, we consider that multiple drugs can have an effect on the genes associated with diseases.

### Prioritization of genes in drug and disease specific subnetworks

**Figure 3:**
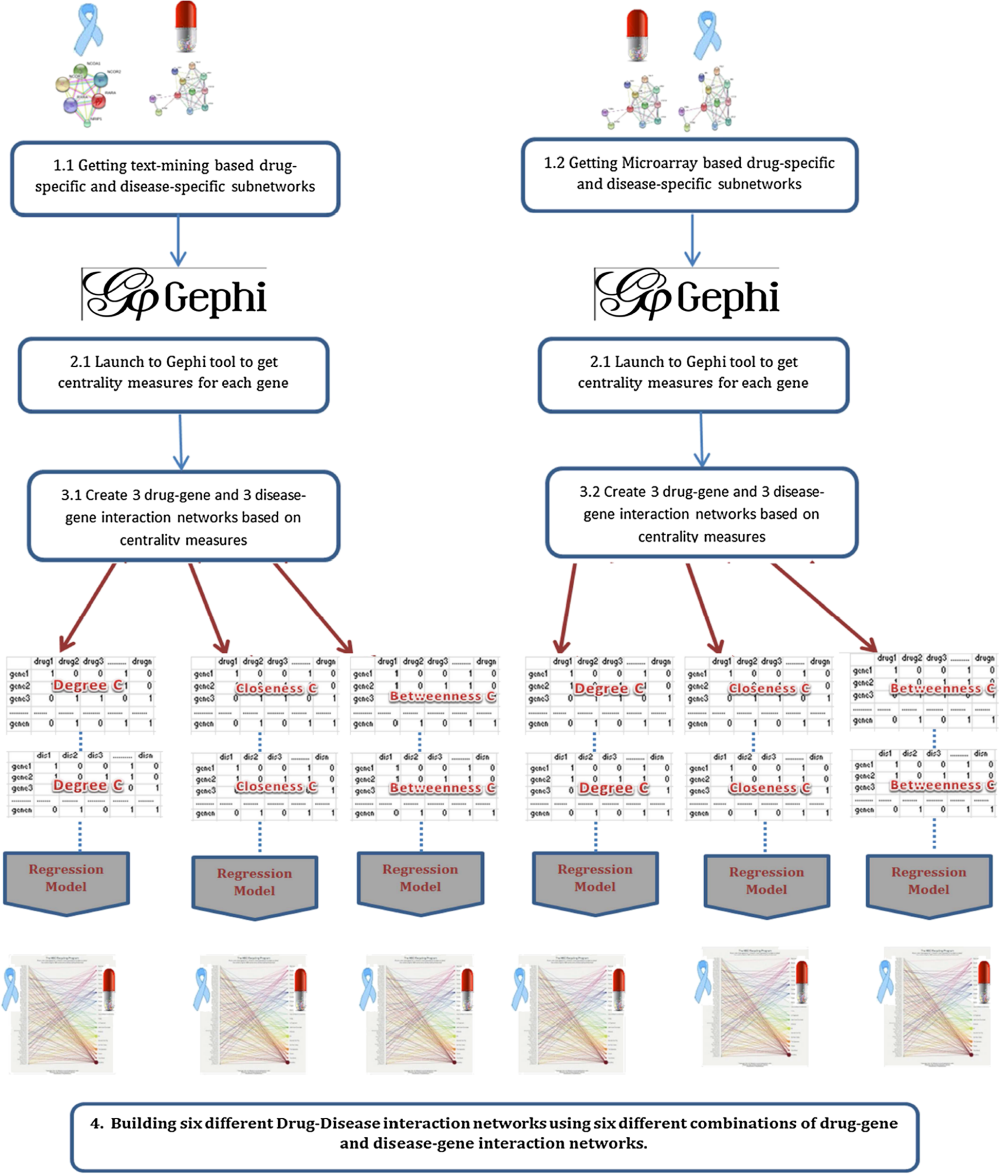
**Building six different drug-disease association networks.** This figure shows the process of building six different drug-disease association networks. Starting with drug-specific and disease-specific subnetworks in step1, we have imported these networks into the Gephi tool to check different centrality measures for every single gene in step 2. In step 3, for each drug/disease we have selected genes with a centrality measure (degree, closeness and betweenness) that is higher than the average centrality measures for all genes. These selected genes have been used to build drug-gene and disease-gene Boolean networks. This step has been independently repeated for the three centrality measures and for the two data sources. Thus we ended up having six different drug-gene and disease-gene networks that have been used to train the regression model in step 4.

After generating the disease-specific and drug-specific gene-gene networks we ran a prioritization process that is based on different centrality measures; namely, degree centrality, closeness centrality and betweenness centrality. The Gephi tool [31] was used to compute these measures for all subnetworks generated for the set of diseases and drugs. Finally for each drug/disease we only considered genes that have a centrality score greater than the average centrality score among all other genes. Thus we built six different drug-gene and disease-gene Boolean interaction networks by utilizing the two subnetworks (text-mining based and microarray based) extracted for each drug and disease. More specifically, for each drug/disease, we used a text-mining based subnetwork to build three drug-gene or disease-gene interaction networks using the three prioritization techniques and did the same to build another three microarray based networks. These networks were then entered into a logistic regression model to produce six different drug-disease interactions as being shown in Figure 3.

### Evaluating the performance of the framework

To evaluate the performance of the integrative framework, we constructed a gold standard disease-drug network from PolySearch server [18]. This gold standard contains 474 positive interactions between 22 diseases and 406 drugs. To generate negative interactions, we selected 400 interactions between disease and drugs that have 0 co-occurrence in PubMed abstracts. We compared the performance of each drug subnetwork to predict disease subnetworks with the gold standard. We used Receiver Operating Characteristics (ROC) curve analysis to produce AUC values to assess the performance of each subnetwork.

## Results

### Selecting a robust set of genes for drugs and diseases

We first selected 571 genes that are targeted by at least one drug in the DrugBank database. We also extracted 820 genes that are associated with a disease according to the OMIM database. To refine these two sets of genes, we incorporated protein networks at this stage. Therefore the final list contained 2343 genes. To prioritize these lists of genes for each drug and disease, we followed two approaches. The first approach prioritized the genes based on their differential expression behavior in cells treated with the drug or in disease samples compared to normal samples. Only the top 50 (25 most upregulated and 25 most downregulated) were selected at this stage. The second prioritization method was based on co-occurrence rate between drugs and genes or disease and genes in PubMed abstracts. Drug-gene and disease-gene pairs with high co-occurrence rate were filtered at the next stage.

### Constructing disease-gene and drug-gene interactions

To predict interactions between diseases and drugs, we first built functional interactions between diseases and genes from one side and drugs and genes from another side. To build each network we followed a systematic integrative approach that incorporates protein networks at several steps in the methodology. We built drug-gene interactions using both text mining (TxtNet-DrugExt) and microarray data (MirNet-DrugExt). Similarly, we built disease-gene interactions using text-mining (TxtNet-DiseaseExt) and microarray (MirNet-DiseaseExt). For the microarray based networks, we incorporated functional protein networks to extract the gene interacting with the top 50 genes representing each drug and disease. As a result, for each drug and disease, we obtained a list of functionally interacting genes to represent drug or disease subnetworks. For the text mining based networks, we incorporated a gene-gene network extracted from STRING database, and then extracted genes linked with the genes which? co-occurred with the drugs or diseases. Finally, for each of these networks, we calculated three centrality measures (degree, betweenness, closeness) of the genes in each network and then selected the genes with high centrality measures as described in the previous section. As a result, we ended up with 12 networks: 6 for drugs and 6 for diseases that were used in the logistic regression model. The resulting disease-gene networks are a matrix of 2343 genes and 22 diseases, and the drug-gene network is a matrix of 2343 genes and 406 drugs. Table 1 summarizes the number of the interactions in each matrix.

**Table 1:** Summary of the networks we generated for each drug and diseases using different centrality measures

**Drug-gene networks**				
**Name**	**Source**	**Size**	**Number of links**	**Centraility measure**
TxtNet-DrugExt-D	Text mining	2343x406	14375	Degree
TxtNet-DrugExt-B	Text mining	2343x406	21443	Betweenness
TxtNet-DrugExt-C	Text mining	2343x406	19890	Closeness
MirNet-DrugExt-D	Microarray	2343x406	29289	Degree
MirNet-DrugExt-B	Microarray	2343x406	34520	Betweenness
MirNet-DrugExt-C	Microarray	2343x406	15350	Closeness
**Disease-gene networks**				
TxtNet-DrugExt-D	Text mining	2343x22	2297	Degree
TxtNet-DrugExt-B	Text mining	2343x22	2471	Betweenness
TxtNet-DrugExt-C	Text mining	2343x22	1199	Closeness
MirNet-DrugExt-D	Microarray	2343x22	1956	Degree
MirNet-DrugExt-B	Microarray	2343x22	1885	Betweenness
MirNet-DrugExt-C	Microarray	2343x22	1062	Closeness

### Performance assessment of different strategies

**Figure 4:**
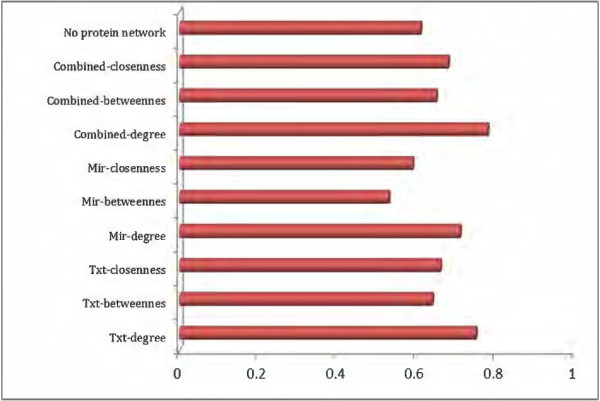
**Performance assessment of different approaches.** This figure shows the performance assessment of multiple approaches to predict disease-drug interactions. Networks that have been generated with genes with a high degree of centrailty have the highest AUC values. Mir-degree is the AUC prediction of using MirNet-DrugExt-D as the predictive variable and MirNet-DiseaseExt-D as the response variable.

After constructing the drug-gene and disease- gene networks, we used logistic regression to predict associations between drugs and diseases, and then assessed the performance of the resulting interaction against the gold standard using AUC. Disease networks were used as response variables and Drug networks were used as predictive variables. Figure 4 shows the AUC values of the six networks described in Table 1. Results show that selecting genes based on their centrality degree in the drug or diseases specific network outperforms other centrality measures. We then combined the text based and microarray based networks for each centrality measure. The results showed that combining text mining and microarray data improves the performance of AUC. When we used protein-based networks to compare the performance of the networks generated to networks that do not incorporate protein, we found that incorporating protein networks improves the AUC as well. This finding also reflects the robustness of networks in revealing some hidden information that can be utilized for prediction purposes.

### Drug-disease network

**Figure 5:**
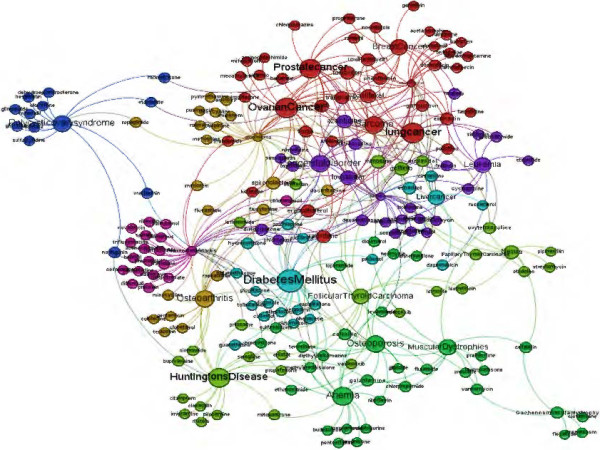
**Predicted drug-disease interaction network.** This figure shows the resulting drug-disease interaction network. Note that different colors represent different drug-disease communities using modularity function in Gephi tool.

A full list of interacting drug-disease networks is available in Additional file 1, which shows the drug-disease network using microarray based networks and using degree centrality. 374 interactions between 22 diseases and 183 drugs are predicted using our proposed regression model. We used the Gephi tool to build a visualized version of these interactions as shown in Figure 5. In the Discussion section we focus on some prostate cancer-drug interactions that were predicted using our proposed paradigm.

### Prostate cancer genes

**Figure 6:**
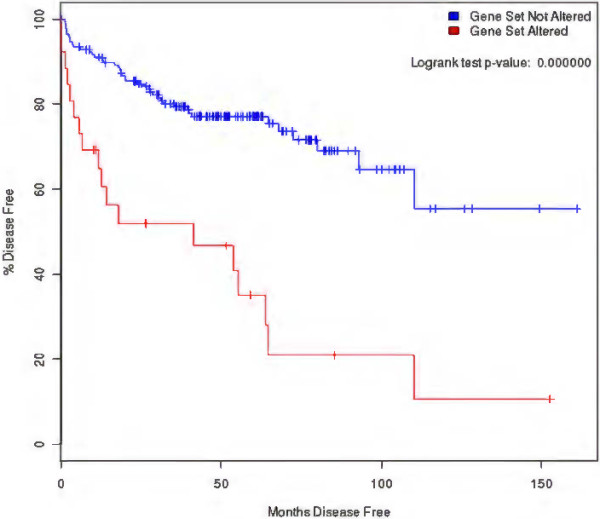
**Kaplan-Meier curve of the 34 prostate genes.** This figure shows the Kaplan-Meier curve of the 34 prostate cancer related genes. From the figure, it can be seen that alteration in these genes is significantly associated with high risk of BCR recurrence.

**Figure 7:**
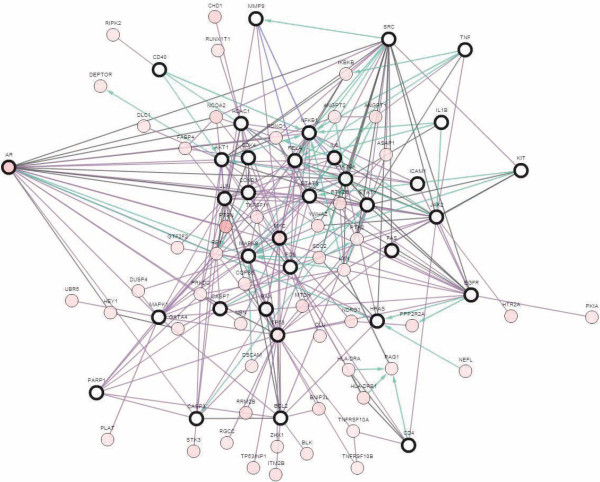
**Protein network of the 34 prostate genes and cancer genes.** This figure shows the functional protein network of the 34 prostate genes predicted by our model and other cancer related protein partners. Most of the genes in this network are oncogenes and tumor suppressors in addition to other keys players in cancer development.

We further assessed the genes found to be relevant to prostate cancer. Based on the microarray-based network, 98 genes were associated with prostate cancer, and based on text mining, 133 genes were associated with prostate cancer; 34 of them were identified with both procedures. We used the Expression2kinase tool to predict drugs targeting those 34 genes, and several were found: e.g., tichostatin, betazole, scriptaid, troglitazone, and felodipine. Unfortunately, none of them was predicted in our approach, due to lack of expression data for these drugs except troglitazone. When we characterized the function of the 34 prostate genes, we found they were significantly associated with BCR free survival (Figure 6) and to multiple cancer pathway genes (Figure 7). These results suggest that the integrative approach we followed to define disease subnetworks to represent each disease can efficiently predict disease related genes. This result provides evidence that predicting drugs that can in effect counteract the 34 genes could be a significant milestone toward reducing prostate cancer risk.

## Discussion

Drug repositioning is one of the most important techniques that is being used to improve drug discovery process. Drug repositioning most attractive feature is its ability to reduce costs and provide shorter paths to approval compared to the daunting traditional techniques. Most of the proposed techniques for drug repositioning tend to use a specific source of data to predict drug-disease interactions. In this work we integrated data from three major sources into a single paradigm to predict some novel drug-disease interactions. More precisely, microarray expression profiles, text-mining and biological networks were all integrated to build a drug-disease network. Comparing the proposed paradigm with a drug-disease gold standard demonstrated the robustness of the integrative paradigm in predicting drug-disease interactions. More specifically, the AUC showed that selecting hub genes from combined network, microarray and text mining, would be more representative than selecting these genes from either of these sources independently. These findings, shown in Figure 4, were validated with considering hub genes in three centrality contexts: degree, betweenness and closeness. The results were consistent in all these centrality measures; hub genes using a combined network were more representative than hub genes using a single data source network. Finally we wanted to check for biological meaning for some of the predicted associations. More specifically we focused on some prostate cancer-drug associations and browsed the scientific literature for biological sense. Azacitidine is a pyrimidine nucleoside analogue that inhibits DNA methyltransferase, impairing DNA methylation [24]. Azacitidine is used for treatment of patients with myelodysplastic syndrome subtypes; refractory anemia with ringed or excess blasts or acute myleogenous leukemia [24]. Azacitidine is believed to exert its effect by causing hypomethylation of DNA on abnormal hematopoitic cells in the bone marrow. According to our study, Azacitidine was found to have a role in prostate cancer treatment. In an experiment to study the effect of Azacitidine in aggressive prostate cancer models, it improved the anti-tumor effect of Docetaxel and cisplatin drugs. The authors suggested using Azacitidine as a chemosensitizing agent in chemoresistant tumors [32]. In another experiment [33], Azacitidine was found to have anti-proliferative activities when administrated chronically. This treatment resulted in a marked decrease in tumor cell proliferation with significant increases in androgen and PSA protein levels. Another interesting association predicted by our suggested model was Berberine and prostate cancer. In many experiments Berberine was found to have anti-tumor activities on prostate cancer cell lines [34,35], and found to induce G1 arrest at low concentration [34]. In addition, at high concentration it has been found to efficiently abrogate G2/M arrest. The results suggest that combined administration of Berberine and caffeine may accelerate the killing of cancer cells. Berberine suppresses AR, which is known to be activated in cancer signaling and suggests that Berberine presents a promising agent for the prevention and/or treatment of prostate cancer [35]. Paclitaxel is an antineoplastic agent indicated as a first-line and subsequent therapy for the treatment of advanced carcinoma of the ovary and other various cancers including breast cancer [24]. According to our model, Paclitaxel was found to have a strong association score with prostate cancer. Indeed, the anti-neoplastic activity of Paclitaxel on prostate cancer was detected in many experiments [36,37]. The findings suggest that Paclitaxel induces nuclear translocation and activation of PKC-Îť, which in turn causes Golgi-Cdk1 activation. Golgi-mediated signaling cascades facilitate mitochondria involved apoptotic pathways, the thing that might explain the anti-tumor activity of Paclitaxel. Surface modified tumor cells may have potential clinical benefit for patients with prostate cancer when it is combined with paclitaxel [35]. With the consideration that immunochemotherapy must depend on careful selection of paclitaxel dosage and the sequence of paclitaxel/vaccine administration.

## Conclusion

The presented results in this work demonstrates that defining robust gene signatures for diseases and drugs from expression profiles and literature and using protein networks to refine and prioritize genes is valuable and have potential in clinical pharmacogenomics research. The results can significantly accelerate the translation into the clinics of known compounds for novel therapeutic uses.

## Competing interests

The authors declare that they have no competing interests.

## Authors’ contributions

AQ conceived and designed the experiments, analyzed the data and wrote the initial manuscript. MA participated in study design, data and results analysis, and wrote initial manuscript. EA participated in protein network analysis. RA participated in study design and data analysis. All authors read and approved the final manuscript.

## Supplementary Material

Additional file 1 Predicted disease-drug interactions. Click here for file
